# Supplementary material for: The Evolution and Characterization of the RNA Interference Pathways in Lophotrochozoa
Source: Genome Biol Evol. 2024 May 7;16(5):evae098. doi: 10.1093/gbe/evae098 (PMC11114477; doi:10.1093/gbe/evae098)
Supplement: evae098_Supplementary_Data [file evae098_supplementary_data.zip › Supplementary_materials.zip!/selected_miRNAs/NoGe_selected_miRNAs/mirdeep_pdf_files/NMRB01000279.1_Notospermus_geniculatus_strain_Ushimado_scaffold279,_whole_genome_shotgun_sequence_9058.pdf]

Provisional ID	:	NMRB01000279.1_Notospermus_geniculatus_strain_Ushimado_scaffold279,_whole_genome_shotgun_sequence_9058
Score total	:	0
Score for star read(s)	:	-1.3
Score for read counts	:	0
Score for mfe	:	1.9
Score for randfold	:	0
Score for cons. seed	:	-0.6
Total read count	:	57
Mature read count	:	49
Loop read count	:	0
Star read count	:	8

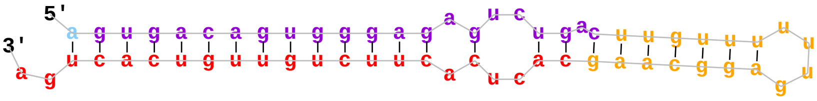

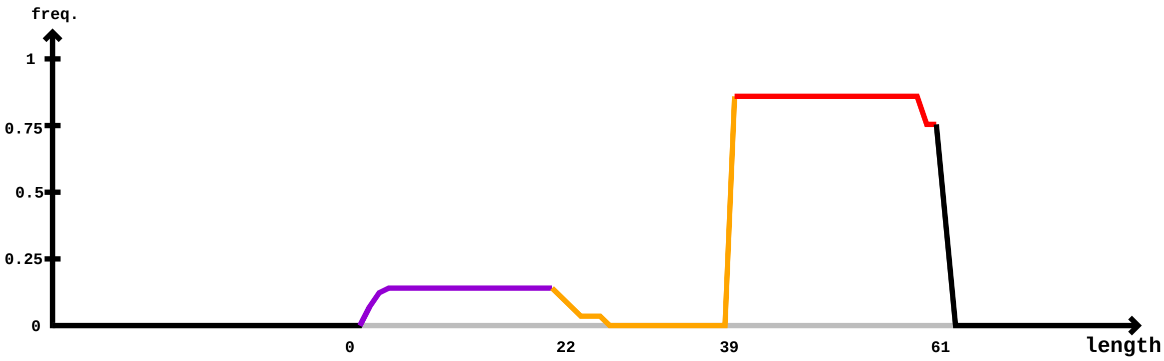

Star Mature

```
5'- gaagccgggcgcagcgaugcgucagccgucagugacagugggagagucugacuuguuuuuugaggcaagcacucacuucuguugucacugaugucuggagagaucgaacaa   -3'   obs
    gaagccgggcgcagcgaugcgucagccgucagugacagugggagagucugacuuguuuuuugaggcaagcacucacuucuguugucacugaugucuggagagaucgaacaa         exp
    ...((..((((((....)))))).))((((((((((((((((((.(..((.(((((((....)))))))))..).))))))))))))))))))(((....)))........    reads   mm   sample
    ...............................gugacagugggagagucugac...........................................................    3       0    Fm3
    ................................ugacagugggagagucugacu..........................................................    3       0    Fm3
    .................................gacagugggagagucugacuugu.......................................................    1       0    Fm3
    .....................................................................cacucacuucuguugucacugU....................    15      1    Fm3
    .....................................................................cacucacuucuguugucacuga....................    5       0    Fm3
    .....................................................................cacucacuucuguugucacCga....................    5       1    Fm3
    .....................................................................cacucacuucuguugucacugUu...................    1       1    Fm3

    .....................................................................cacucacuucuguugucacugU....................    1       1    Fm1

    ...............................gugacagugggagagucugacuugu.......................................................    1       0    Mm2
    .....................................................................cacucacuucuguugucacCga....................    2       1    Mm2

    .....................................................................cacucacuucuguugucacu......................    6       0    Mm1
    .....................................................................cacucacuucuguugucacugU....................    14      1    Mm1
```
